# The Role of Transcriptomics in Redefining Critical Illness

**DOI:** 10.1186/s13054-023-04364-2

**Published:** 2023-03-21

**Authors:** Tiana M. Pelaia, Maryam Shojaei, Anthony S. McLean

**Affiliations:** 1 Department of Intensive Care Medicine, Nepean Hospital, Kingswood, NSW Australia; 2 Centre for Immunology and Allergy Research, Westmead Institute for Medical Research, Westmead, NSW Australia

## Abstract

This article is one of ten reviews selected from the Annual Update in Intensive Care and Emergency Medicine 2023. Other selected articles can be found online at https://www.biomedcentral.com/collections/annualupdate2023. Further information about the Annual Update in Intensive Care and Emergency Medicine is available from https://link.springer.com/bookseries/8901.

## Introduction

Critical care medicine is rapidly evolving, with the approach to sepsis serving as a paradigmatic example. Our understanding of sepsis has been subject to decades of development and refinement, which reflects a continuous effort towards improving the management of this burdensome medical problem. Sepsis was recently redefined as “life-threatening organ dysfunction caused by a dysregulated host response to an infection”, characterizing it as a syndrome that captures a vast heterogeneity of patients [1]. The updated definition is the first to emphasize the primacy of the non-homeostatic host response where the disruption of inflammatory, anti-inflammatory, metabolic, and circulatory processes is driven by a complex array of factors. Transcriptomics, the study of RNA transcripts in a specific cell or tissue, has dramatically progressed alongside critical care medicine, and while there is an inclination to associate key cellular pathways in sepsis with changes in gene expression derived from messenger RNA (mRNA) levels, the role of the transcriptome has expanded tremendously to non-coding RNAs (ncRNA) that possess dynamic regulatory functions.

Despite advancements in the comprehension of its pathophysiology, sepsis remains one of the leading causes of morbidity and mortality in critically ill patients [2]. As reinforced by the Surviving Sepsis Campaign, the current strengths in sepsis management rely on early identification of patients at risk, initial fluid resuscitation, prompt antimicrobial therapy, as well as quickly identifying and controlling theinfection source [3]. Yet due to the notoriety of its heterogeneous manifestations, there is a strong conviction for moving the current treatment paradigm toward a more personalized approach [4–6]. The ultra-sensitivity of transcriptomic profiling systems, such as RNA-sequencing (RNA-Seq), quantitative polymerase chain reaction (qPCR), and microarrays, means that interindividual variability in the host response is provided with a high level of molecular detail. While important insights can be drawn from these tools, the fundamental question is whether they translate to clinical utility. This includes strengthening the existing approach to sepsis that rests on timely intervention, as well as fostering a growing potential to redefine sepsis through the lens of precision medicine. In this chapter, we provide an overview of how RNA participates in sepsis pathophysiology, and give an update on the potential of transcriptomics to uncover new tools in the early detection and treatment of sepsis.

## Transcriptomes: An Indispensable Player in Unraveling the Mechanisms of Sepsis

The growing body of data on sepsis pathophysiology has revealed an unprecedented level of molecular complexity. Such intricate analyses may initially appear to be far removed from the observable clinical characteristics of the critically ill patient. However, it is at this mechanistic level where a profound source of heterogeneity is discovered, providing a fresh outlook on developing rapid and precise methods for managing septic patients. While it is outside the scope of this chapter to investigate the pathophysiology in detail, highlighting the key cellular processes involved assists in understanding the governing role of transcriptomes.

### Overview of the Molecular Pathophysiology of Sepsis

The host response to sepsis begins with detecting the invading microorganism via pathogen-associated molecular patterns (PAMPs). These foreign antigens interact directly with pattern recognition receptors (PRRs) present at the cell surface or intracellularly. This recognition event transduces the pathogenic signal to the cell nucleus through multiple pathways. A core example involves nuclear factor kappa B (NF-κB) signaling, which regulates the transcription of early-activation genes that code for a myriad of pro-inflammatory cytokines. This inflammatory network is crucial for the activation of innate immune cells and subsequent signaling cascades that ultimately serve to eliminate invading pathogens from the host. During early sepsis, however, this response is abruptly upregulated, leading to systemic inflammation that can beget endothelial damage, increased vascular permeability, hypercoagulation and metabolic dysfunction [7]. Reciprocal damage-associated molecular patterns (DAMPs) released from dying cells perpetuate the inflammatory and innate immune response. The secretion of inflammatory mediators is therefore amplified, resulting in sustained tissue inflammation and injury from excessive leukocyte infiltration. End organ dysfunction manifests consequently, with complications like acute respiratory distress syndrome (ARDS), acute kidney injury (AKI), cardiomyopathy, and encephalopathy commonly experienced. Many patients also develop secondary immunosuppression, typically characterized by a concurrent production of anti-inflammatory cytokines to compensate for the overwhelming proinflammatory response. An enhanced anti-inflammatory response is regulated by molecular pathways that result in widespread loss of immune cells and an impaired capacity for antigen presentation [7]. Thus, immunosuppressed patients are subservient to ongoing primary infection, the development of secondary infection, and viral reactivation.

### Messenger RNA: The Driving Force of Transcriptomics

Inherent in the central dogma is the explicit role of mRNAs in sepsis pathophysiology. PRRs, cytokines, signal transducers, and immune cells are all composed of proteins that are coded, and thereby modulated, by mRNA expression. In this way, coding RNA transcripts have substantially informed our understanding of the dysregulated host response, and methods to investigate gene expression have evolved from microarrays that detect a predefined set of sequences, to RNA-Seq that covers the expression of the entire transcriptome. Dynamic gene expression profiles can now be analyzed at the tissue or cellular level, where differentially expressed genes that are up- or down-regulated between defined populations or time points are identified and cataloged to specific biological pathways and functions. In sepsis, transcriptomic studies are typically poised towards analyzing mRNA profiles from peripheral blood leukocytes, but have encompassed cecal ligation and puncture (CLP) animal models, tightly controlled human endotoxemia experiments with healthy volunteers, and clinical studies with critically ill patients that evidently encounter more complexity. The consensus is that the transcriptional response to sepsis is complex and highly protean, with up to thousands of differentially expressed genes emerging simultaneously and progressively [8–10]. Indeed, the transcription of PRR genes, notably those of the Toll-like receptor (TLR) family are upregulated during sepsis, as well as pro-inflammatory cytokines such as tumor necrosis factor α (TNF-α), interleukins (IL)-1α, -1β, -6, and -12, and type-I interferons (IFN) [8, 9]. Pathways associated with signal transduction are also enriched, including NF-κB, mitogen activated protein kinase (MAPK), janus kinase (JAK), and signaling transducer and activator of transcription (STAT) [8–10]. RNA transcripts related to mitochondrial dysfunction, protein synthesis, T helper cell differentiation, endotoxin tolerance, cell death, apoptosis, necrosis, and T-cell exhaustion are also profoundly modulated during sepsis [8, 11]. Novel transcriptional patterns are observed in the dysfunction of various organs, as well as among patients of different sex, age groups, and medical comorbidities [12].

### MicroRNA: The Master Regulators of Gene Expression

There is increasing acknowledgement that a transcriptome-level understanding of sepsis exceeds mRNA expression, with ncRNAs emerging as a prominent feature. In particular, microRNAs (miRNAs) are identified as ‘master regulators’ of gene expression that primarily act post-transcriptionally by interacting with mRNAs to induce mRNA degradation and inhibit translation, and can act intra- and extracellularly [13]. The intricate crosstalk between miRNA and cellular pathways combined with its systemic influence has prompted much research into the involvement of miRNAs in sepsis. Transcriptomic profiling technologies, notably RNA-seq, have been applied to analyze the sepsis-induced effect on miRNAs, and have documented the differential expression of various miRNAs in multiple cell types [13, 14]. These findings have been corroborated with numerous *in vitro* studies to elucidate the function of miRNAs in the immunoinflammatory response, where they are shown to exhibit dynamic pro-inflammatory and anti-inflammatory activities. For example, miR-146a can negatively regulate the TLR4/NF-κB pathway, highlighting its involvement in endotoxin tolerance and attenuating the inflammatory response, thus its downregulation during sepsis worsens inflammation [14]. On the other hand, miR-135a has a pro-inflammatory effect on cardiomyocytes by activating the p38 MAPK/NF-κB pathway, and its expression is elevated in the serum of patients with sepsis-induced cardiac dysfunction [15].

### Long Non-coding RNA: The miRNA Sponges

Long ncRNAs (lncRNAs) were once regarded as transcriptional noise, but their novel roles in gene regulation are now canonical. They have been classified as ‘miRNA sponges’ that bind to and sequester miRNAs, thereby reducing their regulatory effect on mRNAs. This adds another intricate dimension to the transcriptomic mechanisms underpinning sepsis where many lncRNAs are aberrantly expressed [16]. For example, the lncRNA THRIL is upregulated in human bronchial epithelial cells in sepsis and sponges miR-19a, which resulted in increased expression of TNF-α and promoted lung cell apoptosis [17]. Circular RNAs (circRNA) are a novel member of the lncRNA family, with a circular conformation that affords stability and resistance. They too hold the putative function as miRNA sponges, but also as ‘miRNA reservoirs’ that store and transport miRNAs to subcellular locations. Recent studies have elucidated the role of circRNAs in sepsis-induced organ failure via their sponging effects, but this research is still at an early stage [18].

## From Transcriptomics to Clinical Tools

**Fig. 1:**
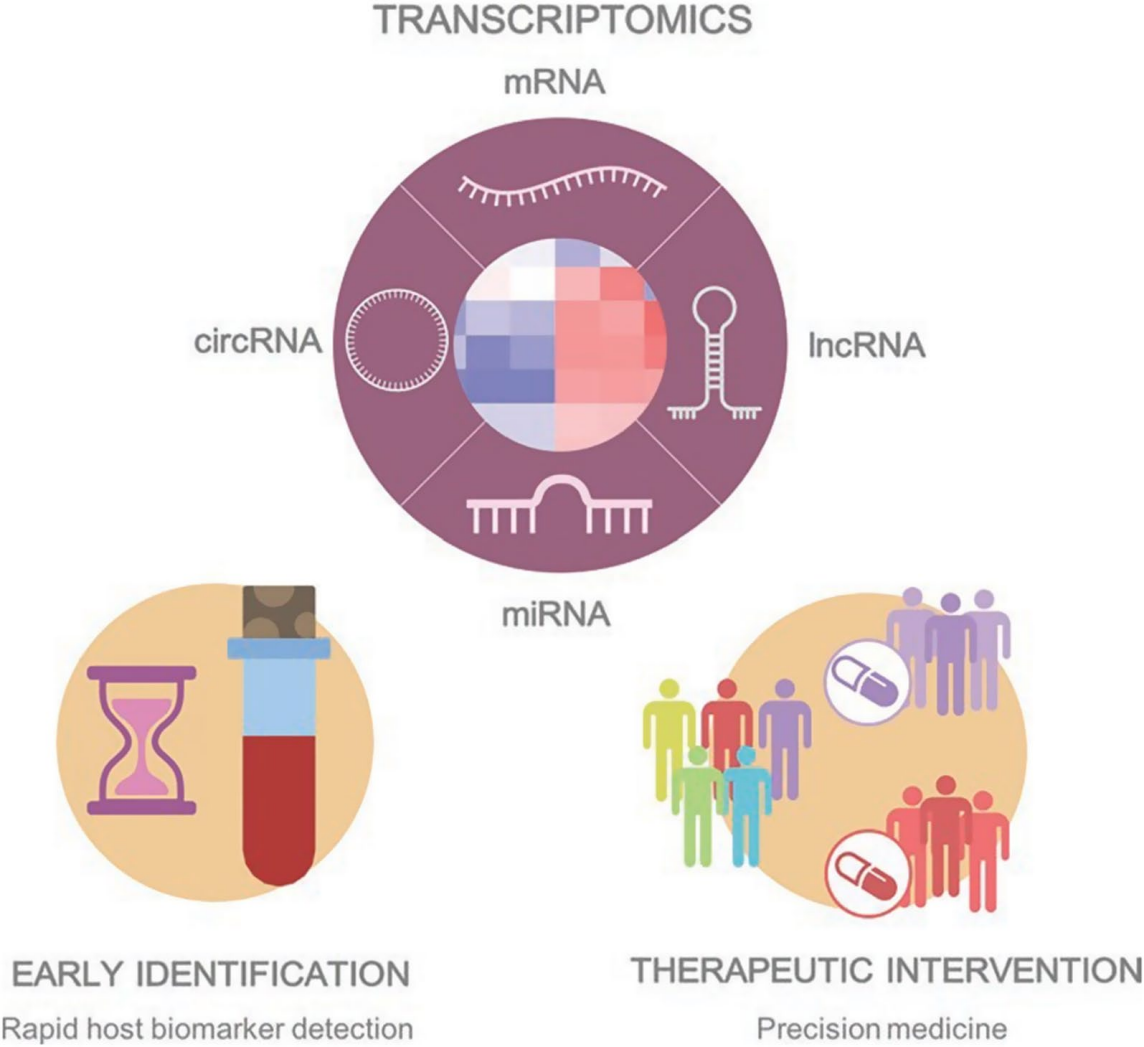
The role of transcriptomics in the early detection of sepsis by developing rapid host biomarkers, and in therapeutic intervention by facilitating a precision medicine approach

Advances in transcriptomics have illuminated three major sources of heterogeneity at the molecular level. First, the cellular functions involved in sepsis are governed by extensive gene regulatory networks involving intricate interactions between mRNAs, miRNAs, and lncRNAs, with the potential to produce a variety of outcomes. Second, expression patterns are highly dependent on the specialized functions of the cell type. Third, the transcriptional response undergoes large dynamic changes as sepsis progresses through different phases, thus giving rise to temporal heterogeneity. The influence of demographic factors and other clinical features adds to this mixed picture, and presents a huge challenge to translate this complexity into clinical practice. Yet with improvements in technologies and clinical trial design, this transcriptomic understanding of sepsis can be sensibly harnessed to address and possibly redefine two fundamental goals of critical care medicine: early identification and effective therapeutic intervention (Fig. 1).

### Time is Critical: Current Challenges in the Early Detection of Sepsis

Sepsis is associated with an increasing risk of mortality for every hour it goes unrecognized, so an early diagnosis is crucial [19]. Ideally, a diagnosis of sepsis should answer the questions that are drawn from its definition: identifying the type of infection, measuring the host response, and predicting the likelihood of organ dysfunction. Identifying the causative pathogen is currently achieved with blood culture, yet a major limitation of this method is the delay to results (typically 48–72 h), which are also frequently read as a false negative [20]. Initial screening tools like the sequential organ failure assessment (SOFA) score can be laborious to calculate in a time-critical emergency, and the use of simplified versions, such as quick SOFA (qSOFA), can be to the detriment of prognostic accuracy [21]. The development of precise and rapid diagnostics is therefore a necessary yet arduous feat in the critical care setting, but biomarker tests for sepsis are emerging as promising candidates. Well established markers such as C-reactive protein (CRP) and procalcitonin (PCT) provide prompt and valuable glimpses into the host response, but discordances in their diagnostic and prognostic performance create the need for a more holistic view of the septic patient [20]. The transcriptomics approach proposes that novel RNA biomarkers can expedite the diagnostic process by harnessing the host response.

### Rapid Host Transcriptomic Biomarkers for Sepsis

The emergence of molecular diagnostics has garnered considerable attention in recent years, whereby rapid qPCR techniques are considered the ‘gold standard’ for detecting novel viruses such as the severe acute respiratory syndrome coronavirus 2 (SARS-CoV-2), yet the same technology can be leveraged for measuring host RNA biomarkers in the blood with fast turnaround times and high accuracy. Several markers warrant specific mention. The *HLA-DRA* gene may be a promising mRNA surrogate of the surface protein HLA-DR on monocytes (mHLA-DR) as a marker of immunosuppression that can be routinely measured with qPCR rather than flow cytometry [22]. miR-150 is a well-investigated miRNA that can discriminate between sepsis and non-infectious systemic inflammatory response syndrome (SIRS) [23]. The lncRNA GAS5 displays prognostic potential in predicting 28-day mortality risk in septic patients [24]. Although far from exhaustive, these individual RNAs reflect the wide-ranging potential of transcriptomics in deriving novel biomarkers for diagnosis and prognostic enrichment. However, a single-biomarker-driven approach towards sepsis is unlikely to be achieved in clinical practice. Many of these biomarkers are only effective at a specific time, in a certain population, or even in a particular tissue or cell, which underscores the perplexity of the sepsis response. Measuring a panel of biomarkers has been advocated to provide greater accuracy and generalizability. As an example, the *IFI27* gene is a well-characterized host biomarker for viral infection [25], but incorporating other viral-induced mRNAs (*JUP* and *LAX1*), as well as mRNAs that are upregulated in bacterial infections (*HK3*, *TNIP1*, *GPAA1*, and *CTSB*) can yield a gene signature that robustly evaluates whether an infection is likely to be of bacterial or viral origin [26]. This 7-mRNA “Bacterial-Viral Metascore” has recently formed part of a composite test alongside an 11-mRNA “Sepsis Metascore” and an 11-mRNA “Stanford Mortality Score” to further affirm the presence of an acute infection and to predict the risk of 30-day mortality (Table 1) [26, 29, 30]. The resultant 29-Host-Immune-mRNA panel called InSep™ (Inflammatix, Bulingame, CA) integrates rapid transcriptomic profiling with advanced machine learning to guide early clinical decisions in the emergency room about administering antibiotics, the need for further diagnostic workup, and the likelihood of an intensive care unit (ICU) transfer [33]. Other groups have reported similar advances in host mRNA expression signatures that have been summarized in Table 1 using areas under the curve (AUCs). Notably, SeptiCyte® RAPID (Immunexpress, Seattle, WA), the first FDA-cleared test to differentiate sepsis from non-infectious SIRS in 1 h, uses host response mRNA expression that is quantified with real time qPCR [28]. It has been clinically validated in retrospective and prospective studies (ClinicalTrials.gov Identifiers NCT01905033, NCT02127502, and NCT05469048). The development of qPCR for host mRNA detection has advanced towards point-of-care devices with the potential to address the unmet need of rapid and early detection of sepsis. Such technologies could also transform the approach to other critical illnesses where a sense of urgency is essential in their management. While the commercial availability of transcriptomic biomarker panels represents an important interface between the bench and the bedside, continued external clinical validation is required to ensure that reproducibility is upheld across heterogeneous populations. The emergence of ncRNA signatures for sepsis diagnosis, including the 14-lncRNA “SepSigLnc”, also gives rise to the possibility of measuring a mixed panel of circRNA, lncRNA, miRNA, and mRNA markers for a more complete and interactive picture of the immuno-inflammatory status [34].

**Table 1:** Host mRNA signatures for the diagnosis and prognosis of sepsis

Setting [Ref]	Transcriptomic score	Performance (validated AUC)	Commercial platform
Sepsis vs. non-infectious SIRS on ICU admission in adults [27]	4-mRNA classifier (SeptiCyte™ LAB SeptiScore™)	0.82–0.89	SeptiCyte™ LAB (Immunexpress, Seattle, WA)
Sepsis vs. noninfectious SIRS in patients with malignancy or treated with antineoplastic/immunosuppressant [28]	Simpler version of SeptiCyte™ LAB (SeptiCyte® RAPID SeptiScore®)	Adult: > 0.88	SeptiCyte® RAPID (Immunexpress, Seattle, WA)
		Pediatric: > 0.96	
Sepsis vs. non-infectious SIRS [29]	11-mRNA classifier (Sepsis MetaScore)	0.83 (0.73–0.89)	Component of the InSep™ test (Inflammatix, Bulingame, CA)
Bacterial vs. viral infection [26]	7-mRNA classifier (Bacterial-Viral MetaScore)	0.91 (0.82–0.96)	Component of the InSep™ test (Inflammatix, Bulingame, CA)
30-day mortality prediction in sepsis patients [30]	12-mRNA classifier (Stanford Score)	0.87 (0.64–1.0)	Component of the InSep™ test (Inflammatix, Bulingame, CA)
28-day mortality prediction in pediatric septic shock [31]	4-mRNA + 12-protein classifier (PERSEVERE-XP)	0.96 (0.91–1.0)	
Abdominal sepsis vs. post-op gastrointestinal surgery control on ICU admission [32]	3-mRNA classifier (sNIP score)	0.91 (0.84–0.97)	

*AUC* area under the curve, *SIRS* systemic inflammatory response syndrome, *ICU* intensive care unit

### Trials and Tribulations: Current Challenges in the Treatment of Sepsis

In a similar vein to diagnosis, therapeutic approaches to sepsis are guided by its definition: controlling the infection, modulating the host response, and ameliorating organ dysfunction. Broad-spectrum antimicrobial therapy is prioritized due to its association in reducing mortality when administered early [3]. Fluid resuscitation and vasoactive agents are essential for the hemodynamic support of vital organ functions. Yet given that the dysregulated host response, rather than the infection itself, is the driver of adverse outcomes, host-directed-therapies have been long-sought-after. After decades of clinical trials, immunomodulatory agents that target PRRs, PAMPs, and pro-inflammatory cytokines have so far proven unsuccessful [35]. This emphasizes the difficulty for preclinical models to fully predict therapeutic efficacy at the bedside where tremendous heterogeneity exists. Attempts have been made to circumvent this challenge by recruiting more homogeneous groups of patients [7]. One study used decreased mHLA-DR levels to stratify sepsis patients for granulocyte-macrophage colony-stimulating factor (GM-CSF) administration, which was found to restore monocyte immunocompetence and shorten mechanical ventilation duration and length of ICU stay [36]. This study, among several others of a similar nature, represent the emergence of a core component of the precision medicine dogma where enrichment strategies are used to identify critically ill patients who could benefit from tailored therapies [6]. Once again, these examples rely on a single biomarker to define patient subsets, which may not capture a holistic view of the complex sepsis response. This is where transcriptomic profiling may facilitate with a more accurate identification of such discrete groups.

### Deriving Transcriptomic Endotypes for Sepsis

As opposed to the top-down prognostic enrichment approach where a clinical feature drives the discovery of transcriptomic signatures associated with it, ‘predictive enrichment’ is a bottom-up approach that is mechanistically driven [6]. Distinct transcriptomic signatures, known as endotypes, are clustered based on shared biological processes that enable the targeted selection of patients who might benefit from targeted host-directed therapies. Several sepsis endotypes have been comprehensively validated and reviewed elsewhere [37], but include the immunosuppressed SRS1 and immunocompetent SRS2 endotypes [11]. Even though they are solely defined by transcriptomic mechanisms, these endotypes show significant differences in clinically relevant characteristics such as 30-day mortality. A post-hoc analysis of the VANISH randomized trial revealed that hydrocortisone administration was associated with higher mortality in the immunocompetent SRS2 endotype compared to the immunosuppressed SRS1, thus serving as an important consideration when designing future prospective trials [38]. While these endotypes were defined according to blood samples collected in the ICU, a recent addition was made to the literature with a multicohort study on emergency room patients with suspicion of sepsis [39]. Patients were stratified into five mechanistically diverse endotypes containing unique ~200-gene signatures denoted as neutrophilic-suppressive (NPS), inflammatory (INF), innate host defense (IHD), interferon (IFN), and adaptive (ADA). Patients with the NPS and IFN endotypes had higher SOFA scores, longer hospital stays, and higher 28-day organ failure. The study employs a theragnostic approach with dual benefit, allowing for the early detection and prognostication of sepsis, and the potential selection of a personalized therapeutic regime. External validation and simpler derivations of these ~200-gene endotypes will be required to improve their clinical utility, before their potential role in informing prospective clinical trial design is realized.

## Challenges of Applying Transcriptomics in Critical Care

Several challenges lie ahead in realizing the full potential of transcriptomics in redefining sepsis and critical care. Peripheral blood has been the pragmatic choice for examining expression patterns, but these profiles may not be accurately extrapolated to other relevant cells involved in sepsis, and important information about specialized cell populations within this mixture may be lost. While methods such as CIBERSORT have been developed to account for leukocyte subtypes in bulk data [40], analyzing a single cell population, whether it be in the blood, the endothelium or from the dysfunctional organ, may be more sensible. The advent of single-cell RNA-seq can help to address this, having to date led to the discovery of novel signatures in monocytes associated with the various immune states [41]. Another challenge involves using transcriptomics to inform and enhance clinical trial design. Personalized approaches that combine prognostic and predictive enrichment strategies have been proposed, whereby patients are stratified based on transcriptomic signatures associated with the likelihood of developing adverse outcomes such as mortality and organ dysfunction (prognostic enrichment), followed by the low-risk patients receiving standard care and the high-risk patients being treated based on their underlying endotype (predictive enrichment) [6]. This leads to another challenge in defining subtypes and signatures that are clinically relevant, molecularly precise, and uniformly applicable. When addressing this, it may be important to realize that transcriptomics is just one dimension of an entire range of modalities that can facilitate a more holistic understanding of the biological pathways in sepsis. An ‘integrated omics’ approach combines data from genomics, epigenomics, transcriptomics, proteomics, lipidomics, metabolomics, and mircobiomics, and can help to build multimodal platforms for diagnosis, prognosis, and drug-discovery. These datasets are open to findings that may address more formidable challenges, particularly in dealing with the rapidly evolving pathophysiology of sepsis. Technological advances that provide clinicians with real-time data at the bedside will also help address this temporal heterogeneity. Importantly, interdisciplinary collaborations between investigators, clinicians, and industry are required to embrace new strategies driven by machine learning and high dimensional data, and to develop cost-effective, rapid technologies that are clinically feasible.

## Conclusion

In this chapter, we have demonstrated the powerful roles of coding and ncRNAs in modulating the septic response. We have highlighted advances in transcriptomics that have enabled the identification of rapid host RNA biomarkers and clinically meaningful endotypes. Early recognition and treatment are the key tenets of current sepsis management, but transcriptomics holds the capacity to view these approaches from a revised angle—one that could facilitate a new era in critical care medicine.

## Data Availability

Not applicable.
